# Progress in Ophthalmology

**Published:** 1895-02-09

**Authors:** 


### PROGRESS IN OPHTHALMOLOGY.

(*Continued from page* 315.)

**The Surgical Treatment of Trachoma.**—Dr. H. Gifford,[4] of Omaha, sounds a note of warning with regard to the use of crushing, scraping, and analogous methods, in the treatment of trachoma, in which he claims to have had a very large experience. He admits that these methods are successful in the great majority of instances, although he thinks that apparent and recorded success is often followed by troublesome relapse, and that the time required for effecting a permanent cure is much longer than is generally supposed. His chief object, however, is to call attention to the occasional occurrence of disastrous results, especially in the presence of corneal ulceration unconnected with the trachoma, and he relates several cases in support of his view. His general conclusion is that no one should use surgical treatment for trachoma unless able to examine the eye with the greatest care and discrimination, and able also, if unusual emergencies should arise, to treat them promptly and vigourously. Without these qualifications, the physician had far better stick to the old blue stone and nitrate of silver treatment; for with these remedies the disease can always be improved or held in check, and in many cases cured; whereas, if he uses surgical measures at all freely, while he may, as a rule, obtain more brilliant results, he is liable, at any time, to ruin an eye which would not otherwise have been lost.

**Scopolamine as a Mydriatic.**—Dr. Harvey Smith,[5] of New York, gives an account of the use of scopolamine as a mydriatic in the Manhattan Eye and Ear Hospital, under the direction of Dr. St. John Roosa. Scopolamine is obtained from the roots of scopolia atropoides, and is isomeric with the other members of the tropeine series. Its physiological and therapeutical effects seem to be almost identical with those of atropine, for which it might in most cases be substituted, but apparently without any particular advantage.

**A New Operation for Glaucoma.**—Nicati,[6] in the "Revue Générale d'Ophthalmologie," has suggested a new operation for glaucoma. A newly-sharpened very narrow knife is introduced, by puncture and counter puncture, into the inferior angle of the anterior chamber, in a manner similar to that employed for sclerotomy, as described by De Wecker. The point of the knife is made to project about a centimetre beyond the counter puncture, the blade is then rotated through ninety degrees, so as to turn its edge towards the iris, which is thus incised as the blade is withdrawn from the eye in its second position. Nicati removes blood from the anterior chamber as completely as possible. No cases are recorded.

**The Treatment of Blepharitis.**—Dr. Essad, in the "Recueil d'Ophthalmologie,"[7] describes a new method of treating blepharitis ciliaris which he has employed for the last two years, with, he says, unvarying success. The treatment, which he gives Despagnet the credit of suggesting, consists in the application of solutions, more or less concentrated, of perchloride of mercury in glycerine. After investigating the condition of the lachrymal apparatus, and correcting any stenosis of the canaliculi, he prescribes two solutions of corrosive sublimate of different strengths. The first contains one part of the perchcloride dissolved in one hundred parts of neutral glycerine. This solution is given to the patient, who is instructed to swab the roots of the lashes daily, taking care not to allow any to pass over on to the conjunctiva, where it would produce a more or less prolonged irritation and feeling of burning, which, however, may be soon allayed by free irrigation with cold water. The second solution, of one part of the perchloride to thirty parts of glycerine, is applied only by the surgeon, and at intervals of two or three days. Before each application all crusts and diseased lashes must be carefully removed, and the application is best made by means of a camel's hair pencil along the edges of the lids, care being taken that none of the solution enters the eye, and any excess being carefully wiped off with a tuft of absorbent cotton wool. If any of the solution should reach the conjunctiva, and occasion smarting, free bathing with cold water must be immediately employed.

**Lactic Acid in Ulcers of the Cornea.**—Dr. Dolgenkow strongly recommends the application of a fifty per cent. solution of lactic acid in the treatment of ulcers of the cornea. The solution is applied by a small pointed stick. The ulcer, so treated, becomes covered with a white crust, which becomes detached in three or four days, leaving a surface in a fair way of cicatrisation. Any healthy portion of cornea which may be accidentally touched is also whitened, but the change does not pass deeper than the epithelium, which is speedily regenerated. The results are said to be excellent; and, in many even severe cases, only a single application of the acid has been required.

[4] Medical Record, Aug. 25, 1894. [5] New York Med. Jour., July 21, 1894. [6] Glas. Med. Jour., July, 1894. [7] New York Med. Chron., Sept., 1894.

